# Rotational changes and associated risk factors following intramedullary nail fixation for intertrochanteric femoral fractures in elderly patients

**DOI:** 10.3389/fsurg.2025.1576336

**Published:** 2025-06-23

**Authors:** Chao Han, Xiaodan Li, Zhe Han, Qiang Dong

**Affiliations:** ^1^ Department of Hip Joint, Tianjin Hospital, Tianjin University, Tianjin, China; ^2^ Department of Anesthesiology, Tianjin First Central Hospital, Tianjin, China

**Keywords:** intertrochanteric fracture of femur, fracture reduction, anteversion change, fracture rotation, internal fixation with intramedullary nail

## Abstract

**Objective:**

To quantify rotational displacement following intramedullary nail fixation for intertrochanteric femoral fractures using three-dimensional (3D) CT imaging, analyze associated risk factors, and evaluate its clinical significance.

**Methods:**

A total of 210 patients who underwent intramedullary nail fixation for intertrochanteric femoral fractures between 2020 and 2022 were included. All patients received postoperative 3D CT scans and were followed for at least 1 year. The range of postoperative femoral anteversion on the affected side was measured, and its correlation with risk factors was analyzed.

**Results:**

Among the 210 participants: six patients exhibited anteversion changes exceeding 15°; seven patients had anteversion changes between 10° and 15°; forty-three patients showed reduced anteversion (indicating increased external rotation of the affected limb), ranging from −1° to −14°, with a mean of −2.58°; in 103 patients, anteversion increased postoperatively, ranging from 1° to 22°, with a mean of 3.90°; sixty-four patients achieved restoration of normal anteversion. Single-nail fixation, poor reduction quality, medial cortical defects, *T*-score ≤ −2.5, Singh's index (Ⅰ–Ⅲ), lateral wall thickness, and insufficient tip-apex distance (TAD) were identified as the primary factors contributing to rotational displacement exceeding 3°. At the 1-year follow-up, patients with smaller rotational displacement demonstrated better functional recovery. The multivariate logistic regression analysis demonstrated that several factors showed significant correlations with rotational displacement of fracture fragments after intramedullary nail fixation, including the severity of medial cortical defects, presence of medial cortical disruption, *T*-scores ≤ −2.5, low-grade Singh's index classifications (I–III), diminished lateral wall thickness, inadequate TAD, Visual Analog Scale (VAS), and the Harris Hip Score (HHS).

**Conclusion:**

Bone rotation and displacement are frequently observed following intramedullary nail fixation for intertrochanteric fractures. Numerous risk factors are closely associated with these complications. Therefore, meticulous attention to surgical technique is essential to minimize complications and optimize outcomes.

## 1 Introduction

Hip fractures in the elderly, often referred to as “the last fracture of life,” represent a significant global health concern. According to the United Kingdom's National Hip Fracture Database (NHFD), the incidence of hip fractures has risen markedly over the past few decades, particularly in regions of Asia and Europe, with an annual average of 70,000–80,000 cases reported (1). Hip fractures primarily encompass femoral neck fractures and intertrochanteric fractures, with the most common treatment involving fracture reduction on a traction bed followed by internal fixation using plate screws or intramedullary nails (2). The quality of fracture reduction is a critical determinant of patient prognosis (3).

During surgical procedures for intertrochanteric fractures, intraoperative assessment of reduction quality is typically performed using C-arm imaging in anteroposterior and lateral views. However, the detection of rotational displacement of fracture fragments remains challenging due to limitations such as the low resolution of plain radiographs and their two-dimensional nature (4). Consequently, research in this area has been limited. Recent studies by Ramanoudjame (5) and Kim et al. (6) utilized three-dimensional (3D) CT scans to evaluate postoperative changes in femoral anteversion. Their findings revealed that 25%–40% of patients exhibited significant changes in anteversion postoperatively, with most cases demonstrating an increase in anteversion, defined as a change exceeding 15° compared to the healthy side. Further analysis by Karaman et al. (7) highlighted the relationship between rotational displacement of fracture fragments and functions of the lower limb, emphasizing that the femoral rotational malalignment of ≥10° is symptomatic for the patients, and the hip, knee, and patellofemoral joints were affected. Because of the possibly altered joint loadings and biomechanics, these could render patients prone to degenerative joint disease. Achieving optimal mechanical stability is crucial for fracture healing, as it reduces complications such as screw cut-out or nail breakage and facilitates early weight-bearing activities.

Given that the hip joint is a weight-bearing structure, alterations in the mechanical axis and alignment of the lower limb can significantly impair patient mobility and function (8). Therefore, attention to rotational displacement in intertrochanteric fractures is essential. Although rotational displacement is not uncommon in the treatment of intertrochanteric fractures, there is a paucity of research on its impact on functional outcomes and associated risk factors. In this study, we retrospectively analyzed clinical data from patients with unstable intertrochanteric fractures treated with intramedullary nailing at our institution. Our aim was to investigate the patterns and extent of rotational displacement, identify related risk factors, and provide insights to guide clinical practice.

## 2 Material and methods

### 2.1 Study design

This retrospective cohort study was conducted in accordance with the STROBE guidelines (9) and adhered to the principles of the Declaration of Helsinki (2013 revision). The study protocol was approved by the Ethics Committee of Hospital. We retrospectively analyzed patients who underwent intramedullary nailing for intertrochanteric femoral fractures between July 2019 and December 2022.

### 2.2 Inclusion and exclusion criteria

Patients who underwent closed reduction and intramedullary nailing for intertrochanteric fractures between 2019 and 2022 were included. Postoperative 3D CT scans of the hip joints were routinely performed. The inclusion and exclusion criteria were as follows:

#### 2.2.1 Inclusion criteria

1) Age ≥ 65 years.
2) Isolated intertrochanteric fracture treated with closed reduction and intramedullary nailing.
3) Ability to walk independently with full weight-bearing prior to the fracture.
4) Absence of uncontrolled medical comorbidities.
5) Availability of complete preoperative and postoperative imaging data, including x-rays and 3D CT scans.

#### 2.2.2 Exclusion criteria

1) Pathological fractures.
2) The presence of severe medical comorbidities or multiple fractures.
3) Previous history of hip surgery.
4) Bilateral fractures or ipsilateral femoral neck fractures.

### 2.3 Anesthesia, reduction, and internal fixation

All patients were positioned supine under general or epidural anesthesia. Closed reduction was performed using a traction table under C-arm guidance. The reduction technique involved full traction of the affected limb, followed by adduction and internal rotation to the neutral position. For fractures that could not be reduced by traction alone, minimally invasive reduction techniques described by Kim et al. (10) and Aktselis et al. (11) were employed.

1) Four types of cephalomedullary nails were utilized based on fracture morphology and surgeon preference:Stryker Gamma3 U-Blade (Stryker, Portage, MI, USA) in 80 cases
2) AO modified proximal femoral anti-rotation nail (PFNA-II) (Synthes, West Chester, PA, USA): Used in 52 cases
3) Smith & Nephew InterTAN (Smith & Nephew, Memphis, TN, USA): Employed in 43 cases
4) Stryker Gamma3 (Stryker, Portage, MI, USA) in 35 cases.

### 2.4 Postoperative management

From the second postoperative day, patients were encouraged to sit up in bed and initiate lower limb muscle strengthening exercises. By the third postoperative day, patients were permitted to sit on the bedside and perform knee flexion and extension exercises, provided there was no evidence of deep vein thrombosis. Six weeks postoperatively, based on fracture healing progress, patients were advised to begin partial weight-bearing with the assistance of double crutches, gradually progressing to full weight-bearing as tolerated.

### 2.5 Perioperative imaging evaluation of fracture rotation and internal fixation quality

#### 2.5.1 Imaging assessment

**Figure 1:**
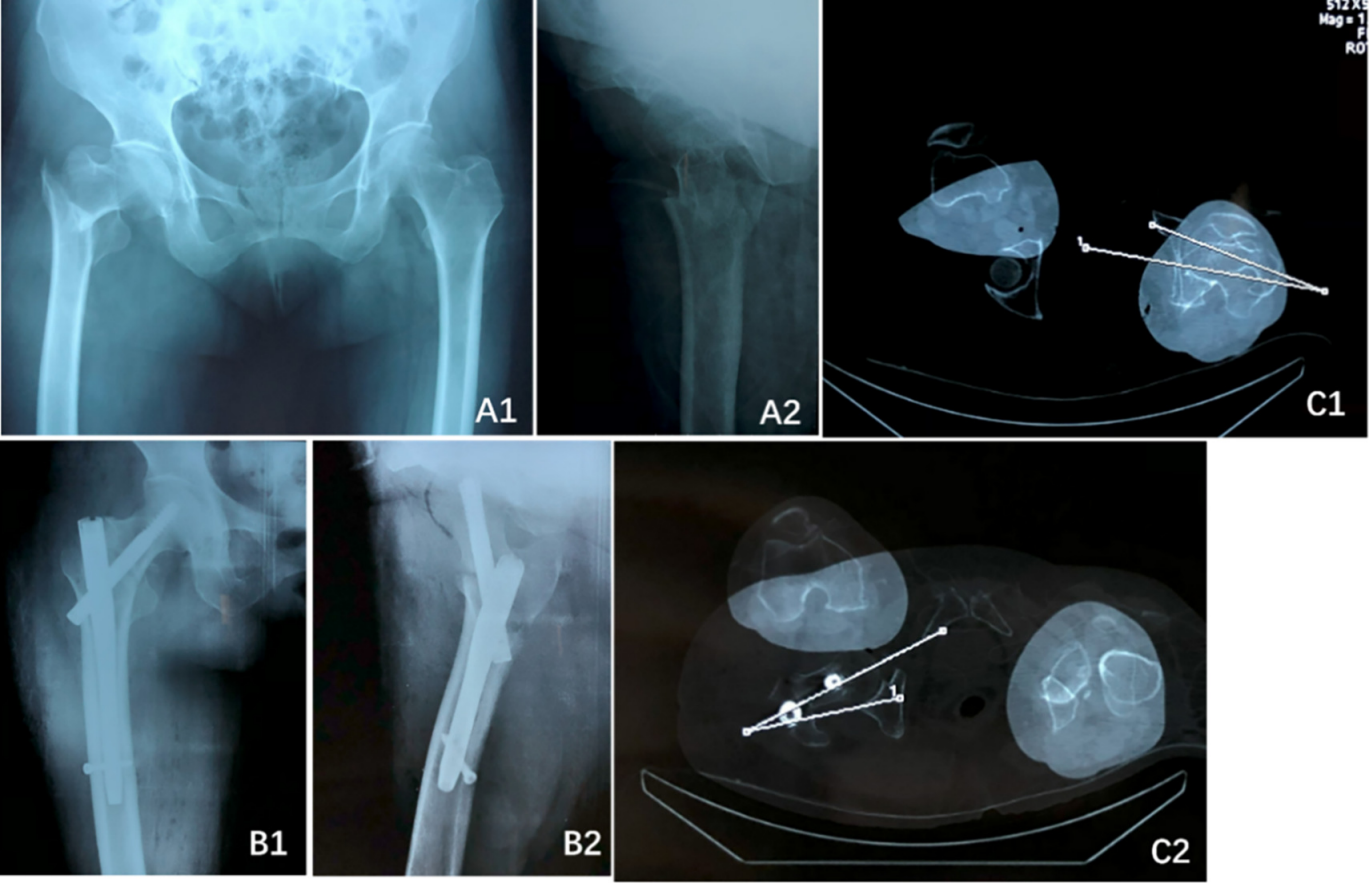
A 75-year-old female patient with a right femoral intertrochanteric fracture (AO/OTA type 31-A2.2). **(A1)** Preoperative anteroposterior (AP) radiograph; **(A2)** Preoperative lateral radiograph; **(B1,B2)** Postoperative AP and lateral radiographs 2 days after surgery, demonstrating excellent fracture reduction and fixation; **(C1)** Measurement of anteversion in the healthy contralateral limb; **(C2)** Measurement of anteversion in the affected limb following internal fixation.

Perioperative imaging, including x-rays and 3D CT scans of the affected hip, was performed to evaluate the rotational displacement of the fracture and assess the quality of internal fixation placement. The change in anteversion of the proximal fracture fragment was measured using the method described by Kim et al. (6). Measurements were conducted by a senior physician using specialized software on a computer, and a second independent measurement was performed by another physician using a protractor on printed films (Figure 1).

#### 2.5.2 Quality assessment of fracture reduction and internal fixation

1. Tip-Apex Distance (TAD) Assessment: The position of the lag screw within the femoral head was evaluated according to the criteria established by Baumgaertner et al. (12).
2. Quality of Internal Fixation: The stability of internal fixation was evaluated by assessing the anteromedial cortical defect of the fracture using 3D CT imaging (13).
3. Fracture Reduction Quality Assessment: The relationship between the proximal bone fragment and the femoral shaft was assessed based on the criteria proposed by Mao et al. (14).

**Figure 2:**
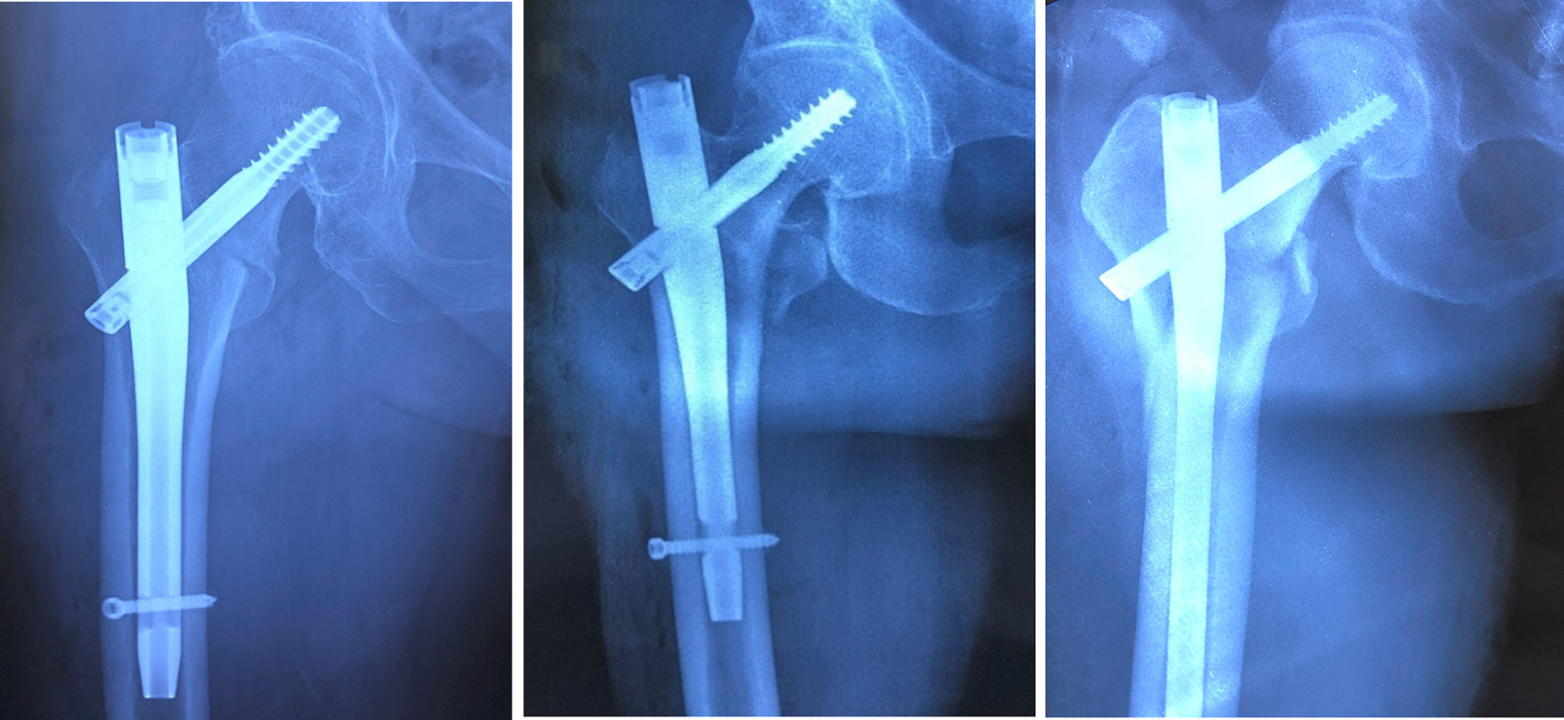
From left to right: positive medial cortical support (PMCS), neutral medial position (NMP), and negative medial cortical support (NMCS).

The medial cortical support was determined from anteroposterior (AP) radiographs and classified as follows (Figure 2):

1) Positive Medial Cortical Support (PMCS): The medial cortex of the head-neck fragment is located superior to the medial cortex of the femoral shaft, with a displacement of less than one cortical thickness.
2) Neutral Medial Position (NMP): The medial cortex of the head-neck fragment is aligned flush with the medial cortex of the femoral shaft.
3) Negative Medial Cortical Support (NMCS): The medial cortex of the head-neck fragment is located lateral to the medial cortex of the femoral shaft, regardless of the degree of displacement.

**Figure 3:**
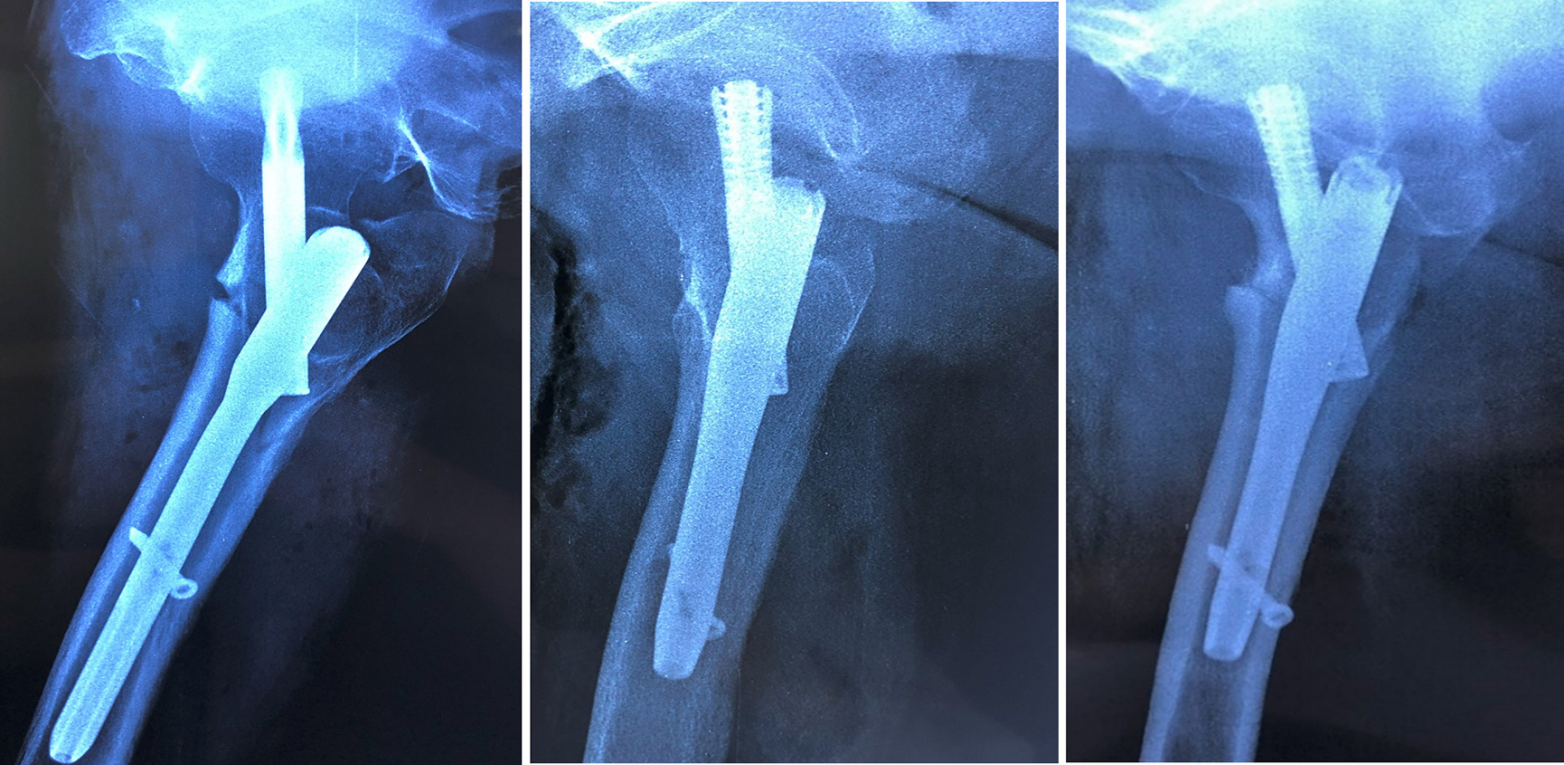
From left to right: positive anterior cortical support (PACS), neutral anterior position (NAP), and negative anterior cortical support (NACS).

The anterior cortical support was assessed from lateral radiographs and classified as follows (Figure 3):

1) Positive Anterior Cortical Support (PACS): The anterior cortex of the head-neck fragment is located anterosuperior to the anterior cortex of the femoral stem, with a displacement of 0.5–1 cortical thickness.
2) Neutral Anterior Position (NAP): The anterolateral cortex of the head-neck fragment is aligned flush with the anterolateral cortex of the femoral stem, or the relative displacement between the two is ≤0.5 cortical thicknesses (regardless of anterior or posterior positioning).
3) Negative Anterior Cortical Support (NACS): The anterior cortex of the head-neck fragment is located posterior to the anterior cortex of the femoral stem, with a displacement exceeding 0.5 cortical thicknesses.

#### 2.5.3 Fracture characterization and osteoporosis assessment

1) Lateral Wall Thickness Measurement: On AP radiographs of the affected hip, the lateral wall thickness was measured at a point 3 cm distal to the tip of the greater trochanter, angled 135° upward to the fracture line (15). In cases of lateral wall fracture, measurements were taken from the contralateral healthy side.
2) Singh Index: The Singh Index, a semi-quantitative morphological grading system, was used to assess trabecular bone loss in the proximal femur based on AP radiographs. Osteoporosis was classified into six grades: Grade VI indicated normal bone density; Grades V and below indicated osteoporosis; and Grades III and below indicated severe osteoporosis (16).

Singh Index grades I–III were classified as severe trabecular bone loss, based on its validated correlation with severe osteoporosis in elderly Asians (17). This simplified categorization (I–III vs. IV–VI) aligns with Asian epidemiologic data where grades IV–VI occur in <5% of fragility hip fractures (18).

3) Osteoporosis *T*-Score: The *T*-score of all patients was recorded. A *T*-score of −2.5 or lower was defined as osteoporosis.

### 2.6 Follow-up and efficacy evaluation

Patients were scheduled for outpatient follow-up 1 year after discharge. Clinical outcomes were assessed using the Visual Analog Scale (VAS) for pain and the Harris Hip Score (HHS) (19), which are widely recognized tools for evaluating pain and functional outcomes in elderly patients with hip fractures.

### 2.7 Statistical analysis

Statistical analysis was performed using SPSS 18.0 software. Normally distributed continuous data were expressed as mean ± standard deviation (mean ± SD), while non-normally distributed data were presented as median (interquartile range, IQR) [M (P25, P75)]. Univariate analysis was conducted using the student's *t*-test or Mann–Whitney *U* test, as appropriate. Categorical variables were analyzed using the chi-square test or Fisher's exact test, depending on the sample size and clinical relevance. Variables with a *P*-value ≤ 0.05 in univariate analysis were included in multivariate analysis using a logistic regression model. A *P*-value ≤ 0.05 was considered statistically significant.

## 3 Results

### 3.1 General results

The study included 210 patients with a mean age of 76.2 years (range: 65–94 years). Among them, 115 were female and 95 were male. The most common injury mechanism was a fall from a standing height. All patients underwent closed reduction and intramedullary nail fixation under C-arm fluoroscopy guidance. The operative duration ranged from 60 to 120 min, with a mean duration of 79 ± 12.5 min.

### 3.2 Characteristics of anteversion changes

The anteversion angle of the healthy hip ranged from 5° to 28°, with a mean value of 14.22°. On the affected side, the anteversion angle ranged from −14° to 22°, with a mean of 1.76°. Among the patients:

1) 6 patients (2.8%) exhibited an anteversion change exceeding 15°.
2) 7 patients (3.3%) had an anteversion change between 10° and 15°.
3) 43 patients (20.4%) showed a decrease in anteversion (indicating increased external rotation of the affected limb), ranging from −1° to −14°, with a mean of −2.58°.
4) 103 patients (49.0%) demonstrated an increase in anteversion, ranging from 1° to 22°, with a mean of 3.90°.
5) 64 patients (30.6%) achieved restoration of the anteversion angle postoperatively.

### 3.3 Quality of fracture reduction

Based on the classification criteria proposed by Mao et al. (14), the quality of fracture reduction was assessed as follows:

1) Excellent: Positive or neutral support observed in both AP and lateral radiographs.
2) Good: Negative support in either AP or lateral radiographs, with positive or neutral support in the other view.
3) Acceptable: Negative support in both AP and lateral radiographs.

Among all patients, 127 cases (60.6%) were classified as excellent, 78 cases (37.0%) as good, and 5 cases (2.4%) as acceptable.

### 3.4 Fracture fragment rotation groupings and correlation with risk factors

Given that the postoperative anteversion angle in most patients was approximately ±3°, we categorized patients into two groups: those with rotational displacement >3° and those with rotational displacement ≤3°. Risk factors, including age, gender, type of internal fixation, reduction quality, AO fracture classification, presence of medial cortical defects, *T*-score, Singh's index, lateral wall thickness and TAD, were statistically analyzed (Table 1).

**Table 1:** Correlation analysis of risk factors associated with rotational displacement in fractures.

Variable	Total (*N* = 210)	<3° (*N* = 146, 69.5%)	>3° (*N* = 64, 30.5%)	*P*
Age (year)	71.5 ± 12.2	71.4 ± 13.8	71.1 ± 11.0	>0.05
Gender [No. (%)]				>0.05
Male	95 (45.2)	67 (45.9)	28 (43.8)	
Female	115 (54.8)	79 (54.1)	36 (56.2)	
Type of lag screw [No. (%)]				0.013*
Single	78 (58.7)	46 (31.5)	32 (50.0)	
Non-single	132 (41.3)	100 (68.5)	32 (50.0)	
*T*-score [No. (%)]				0.007*
>−2.5	88 (41.9)	70 (47.9)	18 (28.1)	
≤−2.5	122 (58.1)	76 (52.1)	46 (71.9)	
Singh's index [No. (%)]				0.000*
Ⅳ–Ⅵ	125 (58.7)	103 (31.5)	22 (50.0)	
Ⅰ–Ⅲ	85 (41.3)	43 (68.5)	42 (50.0)	
Lateral wall thickness (mm, *x¯* ±* s*)	20.76 ± 2.27	21.64 ± 2.21	19.43 ± 1.66	0.000*
Quality of reduction [No. (%)]				0.050*
Excellent	127 (60.5)	89 (61.0)	38 (59.4)	
Good	78 (37.1)	56 (38.4)	22 (34.4)	
Acceptable	5 (2.4)	1 (0.06)	4 (6.2)	
AO classification [No. (%)]				>0.05
Type A1	52 (24.8)	31 (21.2)	21 (32.8)	
Type A2	132 (62.9)	96 (65.8)	36 (56.3)	
Type A3	26 (12.3)	19 (13.0)	7 (10.9)	
Medial cortical defects				0.000*
Yes	71 (33.8)	47 (32.2)	38 (59.4)	
No	139 (66.2)	100 (67.8)	26 (40.6)	
TAD				0.022*
≤25 mm	125 (59.5)	79 (54.1)	46 (71.9)	
>25 mm	85 (40.5)	67 (45.9)	18 (28.1)	
Harris score		81.0 (75.0, 94.0)	75.0 (68.0, 81.0)	0.00*
VAS score		2 (2, 2)	3 (2, 3)	0.00*

*Statistically significant (*p* ≤ 0.05).

The results indicated that single nail fixation, poor reduction quality, medial cortical defects, *T*-score ≤ −2.5, Singh's index (Ⅰ–Ⅲ), lateral wall thickness and insufficient TAD were significantly associated with rotational displacement >3° compared to the control group (*P* ≤ 0.05).

### 3.5 Efficacy and functional outcomes

All patients were followed up for more than 12 months (mean: 12.2 ± 3.6 months) and received guided functional exercises. No cases of nonunion were observed, and fracture healing time ranged from 2 to 6 months (mean: 2.1 ± 1.5 months). There were no complications such as wound infection or internal fixation failure. At the final follow-up, no cases of nonunion, implant loosening, or revision surgery were reported.

At the last follow-up, the median VAS score for patients with rotational displacement >3° was 3 points, compared to 2 points in the control group, showing a statistically significant difference (*P* ≤ 0.05). Similarly, the median HHS for the rotational displacement >3° group was 82 (IQR: 72.5, 91.5), while the control group achieved a median score of 94 (IQR: 88.0, 98.0). Patients with smaller rotational displacement angles demonstrated significantly better functional recovery (*P* ≤ 0.05) (see Table 1 for details).

### 3.6 Multivariate analysis of fracture fragment rotation and associated factors

Multivariate logistic regression analysis revealed that the medial cortical defects, *T*-score ≤ −2.5, Singh's index (Ⅰ–Ⅲ), lateral wall thickness, insufficient TAD, HHS, and VAS score were significantly associated with the degree of fracture fragment rotation following intramedullary nailing (*P* ≤ 0.05) (see Table 2 for details).

**Table 2:** Multivariate analysis of factors influencing rotational displacement following intramedullary nail fixation for intertrochanteric fractures.

Variables	*B*	S.E	Wals	df	Sig.	Exp (B)
Type of lag screw	−0.745	0.426	3.058	1	0.080	0.475
Quality of reduction	−0.289	0.412	0.490	1	0.484	0.749
Medial cortical defects	1.512	0.439	11.882	1	0.001*	4.535
TAD	1.173	0.435	7.282	1	0.007*	3.230
Harris score	0.051	0.023	5.035	1	0.025*	1.052
VAS score	−1.560	0.278	31.431	1	0.000*	0.210
*T*-score	−1.297	0.450	8.324	1	0.004*	0.273
Singh's index	−1.433	0.417	11.808	1	0.001*	0.239
Lateral wall thickness	0.712	0.129	30.391	1	0.000*	2.038

*Statistically significant (*p* ≤ 0.05).

## 4 Discussion

Rotational displacement of fracture fragments is a common postoperative complication, particularly in long bone fractures such as those of the tibia and femur (20). This displacement not only affects cosmesis but also significantly impairs limb function, necessitating targeted research. Currently, there is limited literature on rotational displacement in intertrochanteric femoral fractures. In 2010, Ramanoudjame et al. utilized 3D CT scanning to assess rotational displacement in intertrochanteric fractures and found that approximately 40% of patients exhibited postoperative rotational displacement exceeding 15°, with excessive internal rotation of the hip being the most prevalent (5). However, this study primarily described the distribution of rotational displacement without exploring associated clinical factors or risk factors.

Our study highlights that rotational displacement following intramedullary nailing for intertrochanteric fractures in elderly patients is influenced by multiple clinical factors and may significantly impact postoperative recovery. Unlike previous studies focusing on younger populations, our research specifically targeted patients aged 65 years or older. Consequently, clinical outcomes in our study may be influenced by factors such as advanced age, severe osteoporosis, and internal fixation failure. Abnormal changes in the anteversion angle of the affected hip postoperatively can lead to gait abnormalities, delayed fracture healing, secondary fractures, and even increased re-fracture rates and mortality in elderly patients (21). In this study, we not only analyzed the incidence of rotational displacement in intertrochanteric fractures but also investigated its correlation with clinical outcomes and risk factors, aiming to provide insights for guiding clinical practice.

In the present study, we observed that only six patients (2.8%) exhibited anteversion changes exceeding 15°, while seven patients (3.3%) had anteversion changes between 10° and 15°. The majority of patients demonstrated postoperative anteversion changes within approximately ±3°, a proportion significantly lower than the 20%–30% reported in previous studies (22, 23). This discrepancy may be attributed to advancements in the understanding and management of intertrochanteric fractures in recent years. On one hand, surgeons now prioritize anatomical over functional reduction; on the other hand, the introduction of novel reduction techniques and devices has significantly improved reduction quality.

Given the high reduction quality in the included patients, we categorized those with rotational displacement exceeding 3° to further analyze the impact of clinical indicators and risk factors on rotational displacement in intertrochanteric fractures. Our findings revealed that single-nail fixation, poor reduction quality, medial cortical defects, and insufficient tip-apex distance (TAD) were the primary contributors to rotational displacement beyond 3°. These results align with previous studies, likely due to the strong association between these factors and fracture instability (7). In stable fractures, the anterior fracture gap visible on lateral radiographs, caused by the pull of the gluteus medius and short external rotators, can often be closed by internally rotating the distal limb, as the posterior cortex acts as a hinge (24). However, in unstable fractures (e.g., four-part or reverse oblique fractures), rotational alignment becomes more challenging due to comminution of the posterior cortex, which disrupts its hinge function and complicates anterior cortical reduction (25).

We further evaluated complications associated with excessive anteversion changes, such as implant failure, nonunion, and gait disturbances leading to secondary fractures, using the Harris Hip Score (HHS) and Visual Analog Scale (VAS) for functional assessment. These complications significantly increase re-fracture rates and mortality in elderly patients, underscoring the necessity and relevance of such studies. Our results demonstrated that patients with rotational displacement exceeding 3° had significantly worse HHS and VAS scores at 1 year postoperatively compared to those with displacement ≤3°. This suggests that excessive rotational displacement adversely affects hip function and quality of life in elderly patients, potentially hindering their return to daily activities and work (26).

The excessive rotation observed in intertrochanteric fractures may be attributed to several factors. Elderly patients often present with multiple comorbidities, necessitating closed reduction and minimally invasive fixation to minimize surgical time and bleeding. However, these techniques may compromise the accuracy of anterior and medial cortical alignment and make intraoperative detection of rotational displacement challenge due to limited fluoroscopy. Additionally, despite satisfactory reduction, fracture displacement or rotation may occur during nail or lag screw insertion due to the wedge effect or other technical factors (27).

This study has several limitations. First, the small sample size and short follow-up period may limit the statistical reliability of the findings. Second, the analysis focused solely on intramedullary fixation, lacking comparative data on extramedullary fixation. Third, the study did not explore the safe range of rotational angles or their impact on fracture healing and functional outcomes.

## 5 Conclusion

Intertrochanteric femoral fractures in elderly patients often involve significant spatial displacement, with rotational displacement frequently going undetected intraoperatively. The severity of medial cortical defects, presence of medial cortical disruption, *T*-scores ≤ −2.5, low-grade Singh's index classifications (I–III), diminished lateral wall thickness and inadequate TAD are key factors contributing to rotational displacement and related complications. Therefore, meticulous attention to rotational alignment and the use of appropriate reduction techniques to achieve anatomical reduction are crucial for improving fracture healing rates and reducing complications such as nonunion and functional impairment.

## Data Availability

The raw data supporting the conclusions of this article will be made available by the authors, without undue reservation.
